# The subgingival cultivable bacteria of Albanian subjects with different periodontal status compared to a similar population of Spanish subjects: a case control study

**DOI:** 10.1186/s12903-022-02121-5

**Published:** 2022-03-23

**Authors:** Gerila Tafaj, Margarita Iniesta, Mariano Sanz, David Herrera

**Affiliations:** 1 ETEP (Etiology and Therapy of Periodontal and Peri-Implant Diseases) Research Group, School of Dentistry, University Complutense of Madrid (UCM), Madrid, Spain; 2 Dental Clinic, School of Dentistry, Albanian University, Tirana, Albania

**Keywords:** Subgingival cultivable bacteria, Periodontitis, Microbiological culture, Albania, Spain

## Abstract

**Background:**

The objective was to qualitatively and quantitatively describe the subgingival cultivable bacteria in Albanian subjects and to compare it with a similar Spanish population.

**Materials and methods:**

Consecutive patients, diagnosed as periodontitis in stages I–II or III–IV, and as periodontally healthy or with gingivitis, were studied clinically and microbiologically by means of microbiological culture, including total anaerobic counts, proportions, and frequency of detection of target species. Outcome variables were analysed by Mann–Whitney, Kruskal–Wallis, ANOVA, ANCOVA and Chi-square tests.

**Results:**

In this cross-sectional study, 83 (Albania) and 90 (Spain) subjects were included. No statistically significant differences were observed between test and control populations regarding demographic variables or smoking habit. Significantly higher total anaerobic counts in the Albanian population (*p* = 0.022) were observed, especially in the periodontal health/gingivitis group (*p* = 0.001). In the test population, the proportions of the cultivable bacteria of *Fusobacterium nucleatum* were significantly lower in both the healthy/gingivitis (*p* = 0.022) and stages I–II periodontitis (*p* = 0.034) groups.

**Conclusions:**

The subgingival cultivable bacteria in both periodontitis and non-periodontitis subjects from Albania showed significantly higher total anaerobic counts and lower proportions of the cultivable bacteria of *F. nucleatum* than a similar population of subjects from Spain.

**Supplementary Information:**

The online version contains supplementary material available at 10.1186/s12903-022-02121-5.

## Background

Periodontal diseases are chronic inflammatory conditions affecting the tooth supporting tissues caused by dental biofilms, but modulated by different patient and environment related risk factors. The pathogenesis of both gingivitis and periodontitis result from an imbalance between the infectious challenge (bacterial pathogens organized in dental biofilms) and the host response [1, 2]. Systemic risk factors will influence the host response by the genetic predisposition and systemic health status of the subject, while the environmental conditions as well as by lifestyle factors may influence both the subgingival microbiota and the host response [2]. For example, there is strong evidence that tobacco smoking is a relevant risk factor for the onset and progression of periodontitis, what contributes to the higher prevalence and severity of periodontitis in smokers [3, 4]. Nutrition has also been reported as a relevant lifestyle factor in the onset and progression of periodontal diseases, although its role as a true risk factor has not yet been established [5].

Environmental and lifestyle factors vary among countries and subject populations from different geographical areas and these changes may influence the aetiology and progression of periodontal diseases in these specific places. In fact, differences in the composition of the subgingival microflora have been demonstrated when sampling subjects with similar clinical characteristics, but belonging to different geographical populations [6]. Whereas it is well established that periodontal pathogens such as *Aggregatibacter actinomycetemcomitans, Porphyromonas gingivalis* and *Tannerella forsythia* have demonstrated a strong association with periodontitis [1], and their relative counts and proportions may be different according to different geographical areas [7, 8]. For example, *A. actinomycetemcomitans*, [9] was reported in high prevalence in the Chinese population, compared to that of European and North American populations. Similarly, in Brazil [10] *A. actinomycetemcomitans* was found in high numbers in both periodontitis and in healthy subjects. In Morocco, subjects diagnosed of periodontitis demonstrated a significantly higher prevalence of *A. actinomycetemcomitans* (35.6%) [11], compared to only 5.7% in periodontitis patients in Spain [12].

These studies therefore suggest that differences in the subgingival microbiota composition may occur in populations with different environmental and lifestyle conditions. In fact, differences in the profiles of the subgingival microbiota were also reported by our research group when comparing periodontitis subjects from Spain and The Netherlands [8], and when comparing periodontitis subjects from Colombia, Chile and Spain [13], or more recently, even comparing subjects between Colombia and Spain [14]. Other authors have postulated that beyond the environmental and lifestyle factors, the genetic background of the subjects may also influence the composition of the subgingival microbiota [6, 15]. In fact, it has been reported ethnicity-specific subgingival microbiomes when comparing two populations sharing a common environment but different genetic background [16].

Albania is a developing country in the western Balkans surrounded by Montenegro, Kosovo, Macedonia and Greece, with a population of approximately 3.1 million people. The drastic change in economic and social political conditions in the last 2 decades has significantly impacted on the socioeconomic and environmental factors that may have an impact on health [17], what may also influence the prevalence and severity of periodontal diseases in this country [18]. Factors such as low socioeconomic status, lack of dental health education and limited access to proper oral-health care in a highly diverse ethnic population may influence the prevalence of periodontal diseases by affecting the composition of the oral microbiota.

Since there are no studies characterising the subgingival microbiota from Albanian subjects, we have designed this case–control study, where consecutive subjects were periodontally diagnosed with the new classification of periodontal diseases [19, 20], and the composition of their subgingival microbiota has been studied using anaerobic culture microbiology. As controls, we have characterised, both clinically and microbiologically, a similar population of Spanish subjects. The working hypothesis is that the clear environmental differences between Albania and Spain will significantly influence the microbial composition of the subgingival cultivable bacteria in subjects with different periodontal health status.

## Materials and methods

### Study design

This study was designed as a cross-sectional observational study and it is reported following the Strengthening the Reporting of Observational Studies in Epidemiology (STROBE) guidelines [21]. The protocol of this investigation was approved by the local ethical committees in the respective countries (references 18/128-E in Spain and 197 in Albania) and considers all aspects of the Helsinki Declaration regarding experimentation involving human.

### Patient sample

Subjects seeking dental attending the Faculty of Dentistry in Tirana (Albanian University, Albania) and the Faculty of Dentistry in Madrid [University Complutense of Madrid (UCM), Spain] were screened between April–May 2018 and March 2020 and registered the socio-demographic characteristics of the patients, such as age, gender, origin, smoking, systemic health, medications, and conditions. Patients included in this study met the following criteria: (1) age between ≥ 30 and ≤ 60. The following exclusion study criteria were also evaluated: (1) having less than 16 teeth; (2) patient with a periodontal abscess or necrotizing periodontal diseases; (3) use of systemic antibiotics in the previous month; (4) patient with relevant systemic diseases (diabetes, polymorphonuclear neutrophil defects, other immune system disorders); (5) pregnant or lactating patient; and (6) patient with current anti-inflammatories, anticonvulsant, calcium channel blockers, or immunosuppressant treatments, or 6 months prior to the sample.

When subjects fulfilled these criteria, they were verbally informed about the study and were asked to participate by signing an informed consent. Upon acceptance, each patient was appointed for the study visits.

### Clinical and radiological examination

The patients received a complete periodontal and radiographic examination, including gingival recession, probing depth (PD), clinical attachment loss (CAL) and bleeding on probing (BoP) [22] using a UNC-15 periodontal probe (HuFriedy, Leinmen, Germany). Plaque index (PlI) [23] was evaluated after rinsing the patient with a plaque disclosing solution containing erythrosine (Plac-Control®, Dentaid, Barcelona, Spain).

After this visit, the included subjects were segmented by their periodontal status in three categories using the following criteria [19, 20]: (1) periodontal health and gingivitis: no CAL, no radiographic bone loss (RBL) and PD ≤ 3 mm, assuming no pseudo-pockets; (2) stages I and II periodontitis: PD 4–5 mm, mostly horizontal RBL and no tooth loss due to periodontal reasons. CAL will be 1–2 mm (stage I) or 3–4 mm (stage II), while RBL affects only the coronal third (< 15% for stage I and 15–33% for stage II); and (3) stages III and IV periodontitis: At least two non-adjacent sites with CAL ≥ 5 mm or reaching the middle third of the root, with PD ≥ 6 mm. Evidence of tooth loss due to periodontal reasons.

### Microbiological procedures

#### Microbiological sampling

Samples were taken with two consecutive standardized 30# sterile paper points (Maillefer, Ballaigues, Switzerland). Paper points were inserted into the crevice or pocket and left in place for 10 s. Prior to sampling, four sites per patient were selected, one in each quadrant. The selected sites were isolated from saliva and supragingival plaque contamination with the use of cotton rolls and compressed air. In periodontal health/gingivitis subjects, subgingival samples were taken from the mesio-buccal sites of the first molars and, when absent, from the adjacent second molars (the next alternative was the second premolars and from there, any teeth present mesially). In subjects with periodontitis, subgingival samples were taken from the most accessible site with the deepest PD and BoP, per quadrant. The eight paper points were transferred into a screw-capped vial, containing 1.5 ml of reduced transport fluid (RTF) [24], so an individual pooled sample was obtained from each patient. Samples were sent directly (Spanish samples) or via courier (Albanian samples) to the Laboratory of Research at UCM, Spain, where they were processed within 24–36 h. RTF was the ideal transport medium, as it has been shown to maintain a good viability of anaerobes up to four days after sample collection [24].

#### Direct anaerobic culture

At the Laboratory, samples were homogenized by vortexing for 30 s, and serially diluted in phosphate buffer saline (PBS) (dilutions 10^–1^, 10^–2^, 10^–3^ and 10^–4^). For each sample, 100 µl of at least two of the dilutions were plated on non-selective blood agar medium (Blood Agar Base II, Oxoid, Basingstoke, England), supplemented with haemin (5 mg/l), menadione (1 mg/l) and 5%, sterile horse blood. Plates were incubated for up to 14 days in anaerobic conditions (80% N_2_, 10% CO_2_ and 10% H_2_) at 37 °C. After 7–14 days of anaerobic incubation, suspected colonies were further identified by microscopy, gram-staining and enzyme activity (see Additional file 1: Table S1). The counts of representative colonies (those with colony morphologies compatible with target pathogen morphology) were carried out.

For isolation and quantification of *A. actinomycetemcomitans*, another 100 µl of the 10^–1^ dilution of each sample and 100 µl without dilution were plated onto the selective medium Dentaid-1 [25], that was incubated for 3 days in air with 5% CO_2_ at 37 °C.

### Data analysis

#### Sample size calculation

The outcome variable “proportion of the anaerobic cultivable bacteria of *P. gingivalis*” was selected to calculate the sample size. With a proportion of *P. gingivalis* in Spain of 22.21% [13] and in order to detect a difference in proportions of 16.72% between Albania and Spain, with a 90% of power and a significance of 95%, at least 88 patients per country were necessary. Besides, and to narrow differences between different age groups in different conditions, the overall sample was a uniformed stratified sampling, in which the same size for all the defined categories were assigned. The desired sampling distribution was 30 patients in each category (periodontal health/gingivitis, stages I–II periodontitis, stages III–IV periodontitis), and 10 patients per age cohort, 30–40 years, 41–50 years, 51–60 years, within each periodontal status category.

### Statistical analysis

The statistical unit of the study was the patient. For continuous data, Kolmogorov–Smirnov test and distribution of data were used to assess normality. Data were expressed as means and standard deviations (SD), and as median and interquartile ranges (IQR) for non-parametric data. Categorical data were expressed as percentages.

Demographic data and clinical variables were analysed by Student t test, ANOVA test and chi-square test with probability values adjusted with the Bonferroni correction. For microbiological outcome variables, total anaerobic counts were calculated on blood-agar plates and expressed in total colony-forming units/ml (CFU/ml). Counts for each specific bacterial species, as well as their percentage of total cultivable bacteria, were also calculated for each patient. Counts and proportions were calculated considering all samples. The logarithmic transformation of CFU of bacterial counts was designed to normalise the data distribution. Microbiological variables were compared by t test and ANOVA test, for parametric data, and U Mann–Whitney test or Kruskal–Wallis test with Dunn–Bonferroni post hoc tests, for non-parametric data. Differences between the two countries were further explored by analysis of covariance (ANCOVA), with country as the factor, and PlI and PD were entered into the model as co-variates. In this case, ANCOVA model adjusted means and confidence intervals (CI) were calculated. Proportions of target pathogens were log-transformed to achieve homogeneity of variances.

For categorical data, chi-square test was used, with Bonferroni correction for multiplicity when was necessary.

All statistical analyses were performed using SPSS 20 program package (SPSS Inc, Chicago, IL, USA) and the level of significance was set in 0.05.

## Results

A total of 186 patients were initially recruited (98 in Spain and 88 in Albania). Eight subjects in Spain and five in Albania were excluded due to technical issues in sample transportation and bacteria culturing. A total of 173 subjects, 90 in Spain and 83 in Albania, were recruited, with a similar distribution within the pre-established categories: periodontal health/gingivitis (55 in total, 30 in Spain and 25 in Albania), stages I–II periodontitis (58, 30 and 28, respectively) and stages III–IV periodontitis (60, 30 and 30, respectively). As depicted in Table 1, there were no statistically significant differences between these populations, either for age and gender or among the periodontal categories. Similarly, the percentage of smokers was not significantly different.

**Table 1:** Description of the population characteristics, and comparison according to periodontal status and country

	Comparison		Age			Gender			Smoking		
	First	Second	n	Mean (SD)	*p* value	Female n (%)	Male n (%)	*p* value	Non smokers n (%)	Smokers n (%)	*p* value
Periodontal status group	Health/gingivitis		55	43.00 (8.54)	0.159^a^	32 (58.2)	23 (41.8)	0.286^b^	46 (83.6)	9 (16.4)	0.381^b^
	Periodontitis I–II		58	44.05 (8.77)		33 (56.9)	25 (43.1)		43 (74.1)	15 (25.9)	
	Periodontitis III–IV		60	46.03 (8.49)		29 (48.3)	31 (51.7)		46 (76.7)	14 (23.3)	
Country		Spain	90	45.42 (7.98)	0.107^c^	49 (54.4)	41 (45.6)	0.976^d^	68 (75.6)	22 (24.4)	0.412^d^
		Albania	83	43.30 (9.23)		45 (54.2)	38 (45.8)		67 (80.7)	16 (19.3)	
Periodontal status by country	Health/gingivitis	Spain	30	44.90 (7.47)	1.000^a^	16 (53.3)	14 (46.7)	1.000^b^	25 (83.3)	5 (16.7)	1.000^b^
		Albania	25	40.72 (9.31)		16 (64.0)	9 (36.0)		21 (84.0)	4 (16.0)	
	Periodontitis I–II	Spain	30	45.47 (8.69)		16 (53.3)	14 (46.7)		22 (73.3)	8 (26.7)	
		Albania	28	42.54 (8.75)		17 (60.7)	11 (39.3)		21 (75.0)	7 (25.0)	
	Periodontitis III–IV	Spain	30	45.90 (7.97)		17 (56.7)	13 (43.3)		21 (70.0)	9 (30.0)	
		Albania	30	46.17 (9.12)		12 (40.0)	18 (60.0)		25 (83.3)	5 (16.7)	

n, number of patients; SD, standard deviation

^a^One-way ANOVA test

^b^Chi-square test with Bonferroni correction

^c^Student t test

^d^Chi-square test

Table 2 depicts the clinical variables in the recruited patients within the three established categories. Overall, subjects from Spain showed a statistically significant higher mean PD (*p* = 0.025). PD was significantly higher in Spanish subjects in stages III–IV periodontitis (*p* < 0.001) and PlI in the periodontal health/gingivitis group (*p* < 0.001). Conversely, PlI was significantly higher in stages III–IV periodontitis (*p* = 0.036) for Albanian population.

**Table 2:** Comparison of clinical parameters among periodontal status groups and between countries

		Comparison		n	Mean (SD)	Mean difference	95% CI		*p* value
		First^a^	Second^b^				Lower bound	Upper bound	
PD (mm)	Periodontal status group	Health/gingivitis		55	2.13 (0.30)	− 0.77*	− 1.03	− 0.51	< 0.001
		Periodontitis I–II		58	2.91 (0.46)	− 0.81^†^	− 1.06	− 0.56	< 0.001
		Periodontitis III–IV		60	3.72 (0.78)	− 1.58^‡^	− 1.83	− 1.33	< 0.001
	Country		Spain	90	3.08 (0.90)	0.28^§^	0.03	0.54	0.025
			Albania	83	2.79 (0.77)				
	Periodontal status by country	Health/gingivitis	Spain	30	2.20 (0.31)	0.14^§^	− 0.01	0.31	0.072
			Albania	25	2.05 (0.28)				
		Periodontitis I–II	Spain	30	2.98 (0.47)	0.15^§^	− 0.08	0.40	0.195
			Albania	28	2.82 (0.44)				
		Periodontitis III–IV	Spain	30	4.06 (0.63)	0.67^§^	0.31	1.04	< 0.001
			Albania	30	3.38 (0.78)				
CAL (mm)	Periodontal status group	Health/gingivitis		55	0.25 (0.25)	− 2.80*	− 3.15	− 2.46	< 0.001
		Periodontitis I–II		58	3.06 (0.47)	− 1.56^†^	− 1.89	− 1.22	< 0.001
		Periodontitis III–IV		60	4.62 (1.16)	− 4.36^‡^	− 4.70	− 4.02	< 0.001
	Country		Spain	90	2.70 (1.93)	− 0.00^§^	− 0.59	0.58	0.985
			Albania	83	2.71 (1.98)				
	Periodontal status by country	Health/gingivitis	Spain	30	0.27 (0.25)	0.04^§^	− 0.09	0.18	0.516
			Albania	25	0.22 (0.26)				
		Periodontitis I–II	Spain	30	3.13 (0.46)	0.15^§^	− 0.08	0.40	0.201
			Albania	28	2.98 (0.46)				
		Periodontitis III–IV	Spain	30	4.71 (0.86)	0.17^§^	− 0.43	0.78	0.566
			Albania	30	4.53 (1.41)				
BoP (%)	Periodontal status group	Health/gingivitis		55	9.87 (10.57)	− 25.10*	− 34.69	− 15.51	< 0.001
		Periodontitis I–II		58	34.97 (21.19)	− 29.04^†^	− 38.43	− 19.65	< 0.001
		Periodontitis III–IV		60	64.02 (27.28)	− 54.14^‡^	− 63.66	− 44.63	< 0.001
	Country		Spain	90	37.70 (29.69)	1.33^§^	− 7.85	10.52	0.775
			Albania	83	36.37 (31.53)				
	Periodontal status by country	Health/gingivitis	Spain	30	10.99 (12.94)	2.46^§^	− 3.01	− 7.94	0.370
			Albania	25	8.52 (6.75)				
		Periodontitis I–II	Spain	30	36.62 (21.35)	3.40^§^	− 7.81	14.62	0.545
			Albania	28	33.21 (21.27)				
		Periodontitis III–IV	Spain	30	65.51 (23.30)	2.98^§^	− 11.24	17.20	0.676
			Albania	30	62.52 (31.08)				
PlI (%)	Periodontal status group	Health/gingivitis		55	35.36 (23.52)	− 33.21*	− 44.48	− 21.95	< 0.001
		Periodontitis I–II		58	68.58 (27.35)	− 14.66^†^	− 25.68	− 3.64	0.005
		Periodontitis III–IV		60	83.25 (23.15)	− 47.88^‡^	− 59.05	− 36.71	< 0.001
	Country		Spain	90	64.21 (24.97)	2.29^§^	− 7.41	12.00	0.641
			Albania	83	61.91 (37.74)				
	Periodontal status by country	Health /gingivitis	Spain	30	48.84 (20.88)	29.65^§^	19.97	39.34	< 0.001
			Albania	25	19.18 (14.79)				
		Periodontitis I–II	Spain	30	66.76 (.5724)	− 3.77^§^	− 18.40	10.85	0.607
			Albania	28	70.53 (30.39)				
		Periodontitis III–IV	Spain	30	77.02 (21.31)	− 12.45^§^	− 24.06	− 0.83	0.036
			Albania	30	89.47 (23.58)				

PD, probing depth; CAL, clinical attachment loss; BoP, bleeding on probing; PlI, plaque index; n, sample size; SD, standard deviation; CI, confidence interval

^a^One-way ANOVA test

^b^Student t test

*Periodontal health/gingivitis group versus periodontitis I–II group

^†^Periodontitis I–II group versus periodontitis III–IV group

^‡^Periodontal health/gingivitis group versus periodontitis III–IV group

^§^Spain versus Albania

Table 3 presents the detection of different bacterial species in subgingival samples of Albanian population. The most prevalent bacterial species in periodontal health/gingivitis, stages I–II and stages III–IV periodontitis groups were *Fusobacterium nucleatum* (92%, 92.9% and 86.7%, respectively), *P. gingivalis* (68%, 82.1% and 80%, respectively), *Prevotella intermedia* (52%, 71.4% and 76.7%, respectively) and *Eikenella corrodens* (46.7% in stages III–IV). Statistically significant differences between subjects with and without periodontitis were only found for *P. intermedia* (*p* = 0.048) and *E. corrodens* (*p* = 0.035). In addition, *E. corrodens* was detected with a higher frequency in stages III–IV than in periodontal health/gingivitis (*p* = 0.016). In terms of counts and proportions, comparing subjects with and without periodontitis, statistically significant differences were detected for *P. gingivalis* (*p* = 0.002 for counts and *p* = 0.016 for proportions), *P. intermedia* (*p* = 0.002 and *p* = 0.020, respectively) and *E. corrodens* (*p* = 0.014 and *p* = 0.043, respectively). In the analysis by periodontal status, significant differences were only observed in counts for *P. gingivalis* (*p* = 0.005), *P. intermedia* (*p* = 0.005) and *E. corrodens* (*p* = 0.020), between periodontal health/gingivitis and stages III–IV periodontitis. On the other hand, statistically significant higher counts and proportions of *Actinomyces odontolyticus* were detected in patients without periodontitis (*p* = 0.030 and *p* = 0.030, respectively), but such differences were not maintained in the analysis by periodontal status.

**Table 3:** Detection of subgingival bacterial species in the Albania population

		Health/gingivititis n = 25	Periodontitis I–II n = 28	Periodontitis III–IV n = 30	Differences among groups	Health/gingivitis versus periodontitis
		Mean (SD)	Mean (SD)	Mean (SD)	*p* value	*p* value
*Aggregatibacter actinomycemcomitans*	Counts	0.19 (0.96)	0	0.46 (1.42)	0.208^a^	0.831^c^
	Proportions	0.14 (0.74)	0	0.00 (0.01)	0.215^a^	0.851^c^
	Frequency^e^	1 (4.0)	0 (0.0)	3 (10.0)	0.270^b^	1.000^d^
*Porphyromonas gingivalis*	Counts	2.98 (2.36)	4.42 (2.28)	4.68 (2.50)	0.005	0.002
	Proportions	8.19 (18.49)	12.75 (17.34)	15.18 (23.08)	0.053	0.016
	Frequency	17 (68.0)	23 (82.1)	24 (80.0)	0.314	0.195
*Prevotella intermedia*	Counts	1.99 (2.17)	3.30 (2.30)	3.84 (2.31)	0.005	0.002
	Proportions	5.68 (18.31)	1.91 (4.40)	2.79 (7.48)	0.055	0.020
	Frequency	13 (52.0)	20 (71.4)	23 (76.7)	0.057	0.048
*Tannerella forsythia*	Counts	0.68 (1.61)	1.73 (2.38)	1.05 (2.16)	0.168	0.155
	Proportions	0.07 (0.26)	0.32 (0.46)	0.51 (1.58)	0.113	0.097
	Frequency	3 (12.0)	10 (35.7)	6 (20.0)	0.553	0.121
*Parvimonas micra*	Counts	0.74 (1.74)	0	0.66 (1.73)	0.106	0.237
	Proportions	0.30 (0.64)	0	0.33 (1.10)	0.062	0.094
	Frequency	5 (20.0)	0 (0.0)	4 (13.3)	0.505	0.120
*Fusobacterium nucleatum*	Counts	3.83 (1.52)	4.17 (1.35)	4.11 (1.84)	0.114	0.053
	Proportions	1.65 (2.00)	1.17 (1.46)	2.38 (4.18)	0.487	0.313
	Frequency	23 (92.0)	26 (92.9)	26 (86.7)	0.489	1.000
*Campylobacter rectus*	Counts	0	0.27 (1.01)	0.16 (0.91)	0.393	0.250
	Proportions	0	0.00 (0.03)	0.02 (0.14)	0.393	0.250
	Frequency	0 (0.0)	2 (7.1)	1 (3.3)	0.555	0.550
*Eikenella corrodens*	Counts	0.55 (1.31)	1.41 (2.12)	2.16 (2.41)	0.020	0.014
	Proportions	0.07 (0.17)	0.21 (0.51)	2.65 (12.75)	0.065	0.043
	Frequency	4 (16.0)	9 (32.1)	14 (46.7)	0.016	0.035
*Capnocytophaga* spp.	Counts	0.32 (1.10)	0.12 (0.65)	0.94 (1.93)	0.091	0.550
	Proportions	0.03 (0.11)	0.02 (0.10)	0.27 (0.93)	0.106	0.526
	Frequency	2 (8.0)	1 (3.6)	6 (20.0)	0.135	0.717
*Actinomyces odontolyticus*	Counts	0.37 (1.30)	0	0	0.095	0.030
	Proportions	0.63 (2.19)	0	0	0.096	0.030
	Frequency	2 (8.0)	0 (0.0)	0 (0.0)	0.063	0.088

n, sample size; SD, standard deviation

^a^Kruskal–Wallis test

^b^Chi-square with Bonferroni correction

^c^Mann–Whitney test

^d^Chi-square;

^e^Number and percentage of positive samples

Table 4 depicts the total anaerobic count results of the test and control group analysed together, and by periodontal status and country. Significantly higher anaerobic counts were detected in the periodontitis categories (stages I–II (*p* < 0.001) and III–IV (*p* < 0.001)), compared with the periodontal health/gingivitis group. Higher total counts were observed in Albanian subjects (*p* = 0.022), being this difference statistically significant in the periodontal health/gingivitis group (*p* = 0.001).

**Table 4:** Comparison of total anaerobic counts (log-transformed) among periodontal status groups and between countries

	Comparison		n	Mean (SD)^c^	Mean Difference	95% CI		*p* value
	First^a^	Second^b^				Lower bound	Upper bound	
Periodontal status group	Health/gingivitis		55	6.00 (0.58)	− 0.65*	− 0.91	− 0.40	< 0.001
	Periodontitis I–II		58	6.66 (0.49)	− 0.11^†^	− 0.35	0.13	0.845
	Periodontitis III–IV		60	6.77 (0.58)	− 0.77^‡^	− 1.02	− 0.51	< 0.001
Country		Spain	90	6.38 (0.69)	− 0.22^§^	− 0.41	− 0.03	0.022
		Albania	83	6.61 (0.57)				
Periodontal status by country	Health/gingivitis	Spain	30	5.77 (0.60)	− 0.52^§^	− 0.80	− 0.23	0.001
		Albania	25	6.29 (0.43)				
	Periodontitis I–II	Spain	30	6.66 (0.45)	− 0.00^§^	− 0.25	0.26	0.979
		Albania	28	6.66 (0.54)				
	Periodontitis III–IV	Spain	30	6.72 (0.56)	− 0.10^§^	− 0.41	0.19	0.474
		Albania	30	6.83 (0.60)				

n, sample size; SD, standard deviation; CI, confidence interval

^a^One-way ANOVA test

^b^Student t test

^c^Mean of log-transformed counts

*Periodontal health/gingivitis group versus periodontitis I–II group

^†^Periodontitis I–II group versus periodontitis III–IV group

^‡^Periodontal health/gingivitis group versus periodontitis III–IV group

^§^Spain versus Albania

Tables 5, 6 and 7 depict the subgingival cultivable bacteria (target periodontal pathogens) in both country populations segmented by the three categories. Samples from Albanian subjects were characterized by significantly lower proportions of *F. nucleatum* and *P. intermedia*. In periodontal health/gingivitis and in stages I–II periodontitis, *F. nucleatum* was present in significantly lower proportion (*p* = 0.022 and *p* = 0.034, respectively) in the Albanian samples. The same was true in the category stages III–IV periodontitis for *P. intermedia* (*p* = 0.038). However, when the proportions of *P. intermedia* were adjusted for plaque index and probing depth, as possible confounding factors between countries by an ANCOVA model, this difference between countries was no longer observed (Table 8).

**Table 5:** Counts, proportions and frequencies of detection of target species by country, in periodontal health/gingivitis subjects

	Periodontal health/gingivitis										
	Country	Counts^a^				Proportions^a^				Frequency^b^	
		n	Mean^c^ (SD)	Median (IQR)	*p* value	n	Mean (SD)	Median (IQR)	*p* value	n (%)	*p* value
*Aggregatibacter actinomycemcomitans*	Spain	30	0	0	0.273	30	0	0	0.273	0 (0.0)	0.455
	Albania	25	0.19 (0.96)	0.00 (0.00)		25	0.14 (0.74)	0.00 (0.00)		1 (4.0)	
*Porphyromonas gingivalis*	Spain	30	2.25 (2.39)	1.55 (4.13)	0.276	30	6.74 (14.35)	0.14 (3.87)	0.574	15 (50.0)	0.178
	Albania	25	2.98 (2.36)	3.77 (4.54)		25	8.19 (18.49)	0.12 (4.06)		17 (68.0)	
*Prevotella intermedia*	Spain	30	1.95 (2.12)	1.23 (3.79)	0.943	30	2.80 (8.97)	0.03 (0.57)	0.760	15 (50.0)	0.883
	Albania	25	1.99 (2.17)	2.00 (3.87)		25	5.68 (18.31)	0.00 (0.31)		13 (52. 0)	
*Tannerella forsythia*	Spain	30	0.72 (1.65)	0.00 (0.00)	0.906	30	0.48 (2.10)	0.00 (0.00)	0.563	5 (16.7)	0.715
	Albania	25	0.68 (1.61)	0.00 (0.00)		25	0.07 (0.26)	0.00 (0.00)		3 (12.0)	
*Parvimonas micra*	Spain	30	0.45 (1.40)	0.00 (0.00)	0.511	30	1.44 (6.91)	0.00 (0.00)	0.393	3 (10.0)	0.446
	Albania	25	0.74 (1.74)	0.00 (0.00)		25	0.30 (0.64)	0.00 (0.00)		5 (20.0)	
*Fusobacterium nucleatum*	Spain	30	3.76 (1.41)	4.11 (1.00)	0.584	30	4.55 (5.57)	2.60 (5.22)	0.022	27 (90.0)	1.000
	Albania	25	3.83 (1.52)	4.30 (1.05)		25	1.65 (2.00)	1.09 (1.70)		23 (92.0)	
*Campylobacter rectus*	Spain	30	0.38 (1.17)	0.00 (0.00)	0.107	30	0.13 (0.52)	0.00 (0.00)	0.107	3 (10.0)	0.242
	Albania	25	0	0		25	0	0		0 (0.0)	
*Eikenella corrodens*	Spain	30	0.67 (1.39)	0.00 (0.00)	0.753	30	0.05 (0.14)	0.00 (0.00)	0.821	6 (20.0)	0.741
	Albania	25	0.55 (1.31)	0.00 (0.00)		25	0.07 (0.17)	0.00 (0.00)		4 (16.0)	
*Capnocytophaga* spp.	Spain	30	0.76 (1.56)	0.00 (0.00)	0.215	30	0.35 (1.06)	0.00 (0.00)	0.164	6 (20.0)	0.269
	Albania	25	0.32 (1.10)	0.00 (0.00)		25	0.03 (0.11)	0.00 (0.00)		2 (8.0)	
*Actinomyces odontolyticus*	Spain	30	0	0	0.118	30	0	0	0.118	0 (0.0)	0.202
	Albania	25	0.37 (1.30)	0.00 (0.00)		25	0.63 (2.19)	0.00 (0.00)		2 (8.0)	

n, sample size; SD, standard deviation; IQR, interquartile range; *n* (%), number and percentage of positive samples

^a^Mann–Whitney test

^b^Chi-square test

^c^Mean of log-transformed counts

**Table 6:** Counts, proportions and frequencies of detection of target species by country, in stages I–II periodontitis

	Periodontitis stages I–II										
	Country	Counts^a^				Proportions^a^				Frequency^b^	
		n	Mean^c^ (SD)	Median (IQR)	*p* value	n	Mean (SD)	Median (IQR)	*p* value	n (%)	*p* value
*Aggregatibacter actinomycemcomitans*	Spain	30	0	0	1.000	30	0	0	1.000	0 (0.0)	1.000
	Albania	28	0	0		28	0	0		0 (0.0)	
*Porphyromonas gingivalis*	Spain	30	5.17 (1.31)	5.22 (1.02)	0.431	30	15.96 (19.24)	7.53 (18.83)	0.171	29 (96.7)	0.097
	Albania	28	4.42 (2.28)	5.03 (2.03)		28	12.75 (17.34)	2.02 (21.52)		23 (82.1)	
*Prevotella intermedia*	Spain	30	4.03 (1.61)	4.32 (1.70)	0.532	30	1.98 (3.70)	0.83 (1.49)	0.190	27 (90.0)	0.071
	Albania	28	3.30 (2.30)	4.00 (5.36)		28	1.91 (4.40)	0.26 (1.52)		20 (71.4)	
*Tannerella forsythia*	Spain	30	1.58 (2.28)	0.00 (4.38)	0.748	30	0.31 (0.54)	0.00 (0.67)	0.826	10 (33.3)	0.849
	Albania	28	1.73 (2.38)	0.00 (4.78)		28	0.32 (0.46)	0.00 (0.68)		10 (35.7)	
*Parvimonas micra*	Spain	30	0.17 (0.93)	0.00 (0.00)	0.334	30	0.48 (2.64)	0.00 (0.00)	0.334	1 (3.3)	1.000
	Albania	28	0	0		28	0	0		0 (0.0)	
*Fusobacterium nucleatum*	Spain	30	4.16 (1.96)	4.84 (1.16)	0.233	30	2.93 (3.35)	2.16 (3.61)	0.034	25 (83.3)	0.425
	Albania	28	4.17 (1.35)	4.65 (1.43)		28	1.17 (1.46)	0.53 (1.57)		26 (92.9)	
*Campylobacter rectus*	Spain	30	0.50 (1.55)	0.00 (0.00)	0.632	30	0.94 (4.54)	0.00 (0.00)	0.655	3 (10.0)	1.000
	Albania	28	0.27 (1.01)	0.00 (0.00)		28	0.00 (0.03)	0.00 (0.00)		2 (7.1)	
*Eikenella corrodens*	Spain	30	0.99 (1.85)	0.00 (0.83)	0.429	30	0.20 (0.55)	0.00 (0.00)	0.508	7 (23.3)	0.453
	Albania	28	1.41 (2.12)	0.00 (3.83)		28	0.21 (0.51)	0.00 (0.29)		9 (32.1)	
*Capnocytophaga* spp.	Spain	30	0.75 (1.74)	0.00 (0.00)	0.090	30	0.12 (0.36)	0.00 (0.00)	0.102	5 (16.7)	0.196
	Albania	28	0.12 (0.65)	0.00 (0.00)		28	0.02 (0.10)	0.00 (0.00)		1 (3.6)	
*Actinomyces odontolyticus*	Spain	30	0.12 (0.67)	0.00 (0.00)	0.334	30	0.00 (0.00)	0.00 (0.00)	0.334	1 (3.3)	1.000
	Albania	28	0	0		28	0	0		0 (0.0)	

n, sample size; SD, standard deviation; IQR, interquartile range; *n* (%), number and percentage of positive samples

^a^Mann–Whitney test

^b^Chi-square test

^c^Mean of log-transformed counts

**Table 7:** Counts, proportions and frequencies of detection of target species by country, in stages III–IV periodontitis

	Periodontitis stages III–IV										
	Country	Counts^a^				Proportions^a^				Frequency^b^	
		n	Mean^c^ (SD)	Median (IQR)	*p* value	n	Mean (SD)	Median (IQR)	*p* value	n (%)	*p* value
*Aggregatibacter actinomycemcomitans*	Spain	30	0	0	0.078	30	0	0	0.078	0 (0.0)	0.237
	Albania	30	0.46 (1.42)	0.00 (0.00)		30	0.00 (0.01)	0.00 (0.00)		3 (10.0)	
*Porphyromonas gingivalis*	Spain	30	5.28 (1.93)	5.74 (1.39)	0.559	30	18.86 (20.39)	11.04 (31.23)	0.173	27 (90.0)	0.472
	Albania	30	4.68 (2.50)	5.50 (2.10)		30	15.18 (23.08)	5.04 (16.55)		24 (80.0)	
*Prevotella intermedia*	Spain	30	4.50 (1.97)	5.00 (1.67)	0.285	30	6.79 (12.08)	2.02 (5.57)	0.038	26 (86.7)	0.317
	Albania	30	3.84 (2.31)	4.84 (3.80)		30	2.79 (7.48)	0.43 (2.30)		23 (76.7)	
*Tannerella forsythia*	Spain	30	2.11 (2.49)	0.00 (4.68)	0.099	30	0.65 (1.54)	0.00 (0.67)	0.096	13 (43.3)	0.052
	Albania	30	1.05 (2.16)	0.00 (0.00)		30	0.51 (1.58)	0.00 (0.00)		6 (20.0)	
*Parvimonas micra*	Spain	30	0.32 (1.24)	0.00 (0.00)	0.402	30	0.08 (0.37)	0.00 (0.00)	0.379	2 (6.7)	0.671
	Albania	30	0.66 (1.73)	0.00 (0.00)		30	0.33 (1.10)	0.00 (0.00)		4 (13.3)	
*Fusobacterium nucleatum*	Spain	30	4.29 (1.74)	4.95 (1.33)	0.593	30	2.19 (2.52)	1.24 (2.69)	0.329	27 (90.0)	1.000
	Albania	30	4.11 (1.84)	4.84 (1.44)		30	2.38 (4.18)	0.84 (2.28)		26 (86.7)	
*Campylobacter rectus*	Spain	30	0.64 (1.67)	0.00 (0.00)	0.175	30	0.21 (0.60)	0.00 (0.00)	0.147	4 (13.3)	0.353
	Albania	30	0.16 (0.91)	0.00 (0.00)		30	0.02 (0.14)	0.00 (0.00)		1 (3.3)	
*Eikenella corrodens*	Spain	30	1.07 (2.00)	0.00 (1.00)	0.065	30	0.29 (1.02)	0.00 (0.02)	0.092	7 (23.3)	0.058
	Albania	30	2.16 (2.41)	0.00 (0.00)		30	2.65 (12.75)	0.00 (0.36)		14 (76.7)	
*Capnocytophaga* spp.	Spain	30	1.38 (2.18)	0.00 (4.00)	0.408	30	0.36 (1.00)	0.00 (0.28)	0.398	9 (30.0)	0.371
	Albania	30	0.94 (1.93)	0.00 (0.00)		30	0.27 (0.93)	0.00 (0.00)		6 (20.0)	
*Actinomyces odontolyticus*	Spain	30	0	0	1.000	30	0	0	1.000	0 (0.0)	1.000
	Albania	30	0	0		30	0	0		0 (0.0)	

n, sample size; SD, standard deviation; IQR, interquartile range; *n* (%), number and percentage of positive samples

^a^Mann–Whitney test

^b^Chi-square test

^c^Mean of log-transformed counts

**Table 8:** Comparison of total anaerobic counts and proportions of selected species by adjusting for confounding factors

		Country	n	Mean^a^ (SD)	*p* value^b^	Adjusted mean^c^ (95% CI)	Adjusted* p* value^d^
Total anaerobic counts	Health/gingivitis	Spain	30	5.77 (0.60)	0.001	5.62 (5.42–5.82)	0.000
		Albania	25	6.29 (0.43)		6.46 (6.24–6.69)	

Proportions				Mean (SD)	*p* value^e^	Adjusted mean^f^ (95% CI)	Adjusted* p* value^d^
*F. nucleatum*	Health/gingivitis	Spain	30	4.55 (5.57)	0.022	0.40 (0.20–0.60)	0.040
		Albania	25	1.65 (2.00)		0.05 (-0,17–0.27)	
	Periodontitis I–II	Spain	30	2.93 (3.35)	0.034	0.25 (0.05–0.46)	0.004
		Albania	28	1.17 (1.46)		-0.18 (-0.39–0.02)	
*P. intermedia*	Periodontitis III–IV	Spain	30	6.79 (12.08)	0.038	0.34 (0.44–0.63)	0.078
		Albania	30	2.79 (7.48)		-0.06 (-0.03–0.22)	

n, sample size; SD, standard deviation; CI, confidence interval

^a^Mean of log-transformed counts

^b^Student t test

^c^Adjusted mean of log-trasnformed counts

^d^ANCOVA

^e^Mann–Whitney test

^f^Adjusted mean of log-transformed proportions; ANCOVA model: (a) plaque index and probing depth were entered in the model as co-variates; and (b) to achieve the homogeneity of variances, the proportions of *F. nucleatum* and *P. intermedia* were log-transformed

No statistically significant differences were found between the countries for counts or frequencies of detection of the target pathogens, although some tendencies were observed. In stages I–II periodontitis, samples from Albanian subjects presented less frequently *P. gingivalis* (82.1% versus 96.7%, *p* = 0.097) and *P. intermedia* (71.4% versus 90.0%, *p* = 0.071). Similar findings were observed for *T. forsythia* in stages III–IV periodontitis (20.0% versus 43.3%, *p* = 0.052). Conversely *A. actinomycetemcomitans* (10.0% versus 0%), and *E. corrodens* (76.7% versus 23.3%, *p* = 0.058) were present more frequently in the Albanian population, in stages III–IV periodontitis.

Table 9 depicts the counts and frequencies of detection of target pathogens by clinical categories. Lower counts, proportions, and frequencies of detection of *P. gingivalis* and *P. intermedia* were detected in the periodontal health/gingivitis group, compared with stages I–II (*p* ≤ 0.05) or stages III–IV periodontitis (*p* ≤ 0.05) groups. Similarly, *T. forsythia* was detected in lower counts, proportions, and frequencies of detection in the periodontal health/gingivitis group, compared with stages I–II periodontitis (*p* ≤ 0.05); *F. nucleatum* was detected in lower counts in periodontal health/gingivitis group when compared with stages I–II (*p* = 0.012) or stages III–IV periodontitis (*p* = 0.001); and *E. corrodens* was detected in lower counts in periodontal health/gingivitis when compared with stages III–IV periodontitis (*p* = 0.028). For *Parvimonas micra*, statistically significant higher frequencies of detection were observed in the periodontal health/gingivitis group when compared with stages I–II periodontitis (*p* = 0.045).

**Table 9:** Counts, proportions and frequencies of detection of target bacterial species by periodontal status

	Country	Counts^a^				Proportions^a^				Frequency^b^	
		n	Mean^c^ (SD)	Median (IQR)	*p* value	n	Mean (SD)	Median (IQR)	*p* value	n (%)	*p* value
*Aggregatibacter actinomycemcomitans*	Health/gingivitis	55	0.08 (0.65)	0.00 (0.00)	0.191	55	0.06 (0.50)	0.00 (0.00)	0.194	1 (1.8)	0.243
	Periodontitis I–II	58	0	0		58	0	0		0 (0.0)	
	Periodontitis II-IV	60	0.23 (1.02)	0.00 (0.00)		60	0.00 (0.00)	0.00 (0.00)		3 (5.0)	
*Porphyromonas gingivalis*	Health/gingivitis	55	2.58 (2.38)	3.47 (4.34)	0.000*	55	7.40 (16.02)	0.12 (3.51)	0.000*	32 (58.2)	0.000*
	Periodontitis I–II	58	4.81 (1.86)	5.14 (1.48)	0.564^†^	58	14.41 (18.26)	6.89 (20.03)	1.000^†^	52 (89.7)	1.000^†^
	Periodontitis III–IV	60	4.98 (2.23)	5.70 (1.70)	0.000^‡^	60	17.02 (21.67)	8.41 (24.46)	0.000^‡^	51 (85.0)	0.003^‡^
*Prevotella intermedia*	Health/gingivitis	55	1.97 (2.13)	2.00 (3.85)	0.000*	55	4.11 (13.94)	0.00 (0.50)	0.007*	28 (50.9)	0.003*
	Periodontitis I–II	58	3.68 (1.99)	4.30 (1.75)	0.220^†^	58	1.95 (4.02)	0.63 (1.34)	0.744^†^	47 (81.0)	1.000^†^
	Periodontitis III–IV	60	4.17 (2.16)	4.92 (1.57)	0.000^‡^	60	4.79 (10.17)	0.87 (3.11)	0.000^‡^	49 (81.7)	0.000^‡^
*Tannerella forsythia*	Health/gingivitis	55	0.70 (1.62)	0.00 (0.00)	0.048*	55	0.29 (1.56)	0.00 (0.00)	0.048*	8 (14.5)	0.042*
	Periodontitis I–II	58	1.65 (2.31)	0.00 (4.63)	1.000^†^	58	0.31 (0.50)	0.00 (0.62)	1.000^†^	20 (34.5)	1.000^†^
	Periodontitis III–IV	60	1.58 (2.37)	0.00 (4.48)	0.078^‡^	60	0.58 (1.50)	0.00 (0.51)	0.099^‡^	19 (31.7)	0.090^‡^
*Parvimonas micra*	Health/gingivitis	55	0.58 (1.55)	0.00 (0.00)	0.091	55	0.92 (5.12)	0.00 (0.00)	0.054	8 (14.5)	0.045*
	Periodontitis I–II	58	0.09 (0.69)	0.00 (0.00)		58	0.24 (1.90)	0.00 (0.00)		1 (1.7)	0.342^†^
	Periodontitis III–IV	60	0.49 (1.50)	0.00 (0.00)		60	0.21 (0.82)	0.00 (0.00)		6 (10.0)	1.000^‡^
*Fusobacterium nucleatum*	Health/gingivitis	55	3.80 (1.45)	4.30 (1.00)	0.012*	55	3.23 (4.53)	1.74 (3.16)	0.241	50 (90.9)	0.666
	Periodontitis I–II	58	4.16 (1.68)	4.69 (1.09)	1.000^†^	58	2.08 (2.74)	1.12 (3.05)		51 (87.9)	
	Periodontitis III–IV	60	4.20 (1.78)	4.90 (1.30)	0.001^‡^	60	2.28 (3.43)	1.08 (2.56)		53 (88.3)	
*Campylobacter rectus*	Health/gingivitis	55	0.20 (0.88)	0.00 (0.00)	0.737	55	0.07 (0.88)	0.00 (0.00)	0.787	3 (5.5)	0.566
	Periodontitis I–II	58	0.39 (1.31)	0.00 (0.00)		58	0.49 (3.27)	0.00 (0.00)		5 (8.6)	
	Periodontitis III–IV	60	4.40 (1.35)	0.00 (0.00)		60	0.12 (0.44)	0.00 (0.00)		5 (8.3)	
*Eikenella corrodens*	Health/gingivitis	55	0.62 (1.34)	0.00 (2.25)	0.386*	55	0.06 (0.15)	0.00 (0.00)	0.089	10 (18.2)	0.128
	Periodontitis I–II	58	1.19 (1.98)	0.00 (3.38)	0.832^†^	58	0.20 (0.53)	0.00 (0.02)		16 (27.6)	
	Periodontitis III–IV	60	1.61 (2.26)	0.00 (4.00)	0.028^‡^	60	0.47 (9.04)	0.00 (0.22)		21 (35.0)	
*Capnocytophaga* spp.	Health/gingivitis	55	0.56 (1.38)	0.00 (0.00)	0.059	55	0.20 (0.80)	0.00 (0.00)	0.097	8 (14.5)	0.125
	Periodontitis I–II	58	0.45 (1.35)	0.00 (0.00)		58	0.07 (0.27)	0.00 (0.00)		6 (10.3)	
	Periodontitis III–IV	60	1.16 (2.05)	0.00 (2.70)		60	0.31 (0.96)	0.00 (0.09)		15 (25.0)	
*Actinomyces odontolyticus*	Health/gingivitis	55	0.17 (0.88)	0.00 (0.00)	0.326	55	0.28 (1.49)	0.00 (0.00)	0.326	2 (3.6)	0.137
	Periodontitis I–II	58	0.06 (0.48)	0.00 (0.00)		58	0.00 (0.00)	0.00 (0.00)		1 (1.7)	
	Periodontitis III–IV	60	0	0		60	0	0		0 (0.0)	

n, sample size; SD, standard deviation; IQR, interquartile range; *n* (%), number and percentage of positive samples

^a^Kruskal–Wallis test

^b^Chi-square with Bonferroni correction

^c^Mean of log-transformed counts

*Periodontal health/gingivitis group versus periodontitis I–II group

^†^Periodontitis I–II group versus periodontitis III–IV group

^‡^Periodontal health/gingivitis group versus periodontitis III–IV group

## Discussion

The present study evaluated two similar populations in terms of age, gender and smoking habits, but from two countries with different environments (Albania and Spain). These subjects have provided microbiological samples processed by anaerobic culturing in a single laboratory using the same microbiological diagnostic technology. These recruited subjects were present in similar numbers in the three diagnostic categories, with minimal differences in the clinical parameters (deeper mean PD in Spanish subjects within the stages III–IV periodontitis category, and lower plaque index levels in Albanian subjects in the periodontal health/gingivitis group but higher in the stage III–IV periodontitis). The analysis of the subgingival cultivable bacteria of these two distinct populations has shown that Albanian subjects presented higher anaerobic counts, especially in periodontal health/gingivitis subjects, and lower proportions of *F. nucleatum* in periodontal health/gingivitis and stages I–II periodontitis.

The fact that all samples were processed by the same microbiological laboratory may reduce the differences due to sample processing reported in previous studies with similar objectives [6, 8, 11, 13, 14]. Also, the fact that the clinical parameters within the pre-established clinical categories were based on the diagnostic criteria of the new classification of periodontal diseases [19, 20] may have reduced the likely differences in the subgingival bacteria due to the differences in periodontal status. Although the Spanish patients showed deeper mean PDs and lower PlI levels in stages III–IV periodontitis, and Albanian patients presented a lower PlI in the periodontal health/gingivitis group, these differences may be anecdotal and with a clear lack of clinical significance, except in stages III–IV periodontitis.

In the present study, when evaluating the microbiological profile of the Albanian population, the bacterial species with the highest frequency of detection in periodontitis were *P. gingivalis*, *P. intermedia*, *F. nucleatum* and *E. corrodens*. There is no available information from Albania to compare with the findings. If they are compared with findings from other geographical locations (Spain, Morocco, Colombia, Chile), *P. gingivalis*, *P. intermedia* and *F. nucleatum* are consistently three of the most frequently detected bacterial species in subgingival samples in patients with periodontitis by means of culture techniques [8, 11, 13, 14]. *E. corrodens* has also shown higher frequencies in periodontitis than in periodontally healthy subjects [26]. However, in the Albanian population studied, only *P. intermedia* and *E. corrodens* showed a statistically significantly higher prevalence in periodontitis than in subjects without periodontitis, which may suggest that *P. gingivalis* and *F. nucleatum* are also frequently present in subjects without periodontitis in Albania.

When the Albanian subjects were compared to a similar Spanish population, two main differences were identified, namely total anaerobic counts and the proportions of specific target species. For the differences in total anaerobic counts, statistically significant differences were detected in the whole sample, which corresponds to higher total counts in Albanian patients than in Spanish patients in the group of subjects with periodontal health or gingivitis. This finding is consistent with a previous study observing microbiological differences in subjects according to race/ethnicity, family income or education, as well as smoking, diet and health habits, or access to dental care [27]. While smoking is not a differentiating variable in the present study, socio-economic and/or socio-demographic differences might have influenced the results.

For differences in the proportions of specific target species, significantly lower proportions of *F. nucleatum*, in periodontal health/gingivitis and in stages I–II periodontitis, were detected in Albanian samples. This finding is in line with a study showing that subjects with low socio-economic status and low levels of oral diseases (caries and/or periodontitis) have lower amounts of certain members of the *Fusobacterium* genus [28]. It is unclear whether these differences may reflect that dysbiotic biofilms in Albanian patients were not clearly associated with specific pathogens, while the corresponding dysbiotic biofilms in Spanish subjects were associated with specific pathogens. Previous studies with populations in Spain have highlighted the possible relevant role of *P. gingivalis* [8, 13, 14], what may support the importance of this pathogen as a key-stone pathogen responsible of the bacterial dysbiosis concept [29].

Although not statistically significant, other microbiological differences also showed clear tendencies in terms of frequencies of detection, depicting higher prevalence of *A. actinomycetemcomitans* and *E. corrodens* in Albania, and higher prevalence of *P. gingivalis, P. intermedia* and *T. forsythia* in Spain. These findings support previous reports comparing the subgingival cultivable bacteria of Colombian and Spanish patients [14], that suggests that dysbiotic biofilms could be associated with larger amounts of microorganisms in Albanian subjects, while in Spain the impact of key pathogens may be more relevant. In addition, the role of *A. actinomycetemcomitans* in promoting dysbiosis of in a limited number of Albanian patients should also be considered, which is consistent with the results of different studies on Eastern Europe populations [30–32], as compared with lower levels in Spain [8, 12–14].

When evaluating the microbiological findings within the different periodontal categories, differences were observed in terms of total anaerobic counts, and in counts, proportions and frequencies of detection of target bacterial species, including the most relevant periodontal pathogens*, P. gingivalis, T. forsythia, P. intermedia* and *F. nucleatum*, what is in agreement with previous studies using other classification systems [6, 8, 31] or with studies using the same 2018 classification [14, 32]. Whether these differences are causal or secondary to differences in PDs cannot be explored in a cross-sectional study [33].

The present study used culture techniques for the identification of the cultivable bacteria associated with periodontitis. While Next Generation Sequencing (NGS) approaches are currently frequently used, an initial characterization of a previously not tested population (as Albanian subjects) may benefit from a simpler approach. However, the value of culture techniques should not be underestimated, alone or in combination with other approaches, since it has been considered that it is important to have parallel culture libraries [34, 35], that benefits from the improvement of microbiological culturing, e.g. with the introduction of more competent anaerobic handling and incubation procedures, so culture is reinvented every day [36]. Thus, many other researchers still believe that cultivation continues to be an interesting alternative for microbiological testing [37] and its use allows for appropriate comparisons with previous studies that have also used culture techniques.

The results of the present study should be interpreted with caution due to the clear limitations of the microbiological methodology used, e.g. the microbiological samples from Albania were sent by courier to Spain, and although the same standardised approach to sampling was followed in both centres [38], and the time interval between sampling and plating was the same for both countries (24–36 h), it cannot be discarded that the ideal transport conditions might not have been maintained for some samples, which could have impacted on the viability of some microorganisms [24]. Another limitation is associated with the sampling strategy, since only the four deepest sites were sampled in each patient, which may underestimate detection frequencies [39]; however, this strategy was validated in the early nineties [40, 41] and it has been extensively used in periodontal microbiology. Moreover, the relatively small sample size without providing information about other possible sources of bias (as differences in socio-economic status) may have limited the opportunity to find significant differences. Finally, culture techniques are not able to provide a thorough research of the subgingival microbiota, thus further in-depth analysis, e. g. using NSG approaches, would be necessary to have a more comprehensive picture of the whole microbiota of the Albanian population, including non-culturable bacterial species.

## Conclusions

Within the limitations of the present study, it can be concluded that the microbiological profile of the subgingival cultivable bacteria in periodontitis and non-periodontitis patients has demonstrated statistically significant differences between Albanian and Spanish patients, with higher total anaerobic counts in Albania and higher proportions of cultivable bacteria of *F. nucleatum* in Spain.

## Supplementary Information

**Additional file 1.** Presumptive identification of bacterial species in culture.

## Data Availability

The datasets generated and analyzed during the current study are not publicly available due to data protection regulations and ethical concerns but are available from the corresponding author on reasonable request.

## Abbreviations

UCM: University Complutense of Madrid
PD: Probing depth
CAL: Clinical attachment loss
BoP: Bleeding on probing
PlI: Plaque index
RBL: Radiographical bone loss
RTF: Reduced transport fluid
PBS: Phosphate buffer saline
SD: Standard deviation
IQR: Interquartile range
CFU: Colony-forming units
CI: Confidence interval
n: Sample size
n (%): Number and percentage of positive samples
