# Supplementary material for: The subgingival cultivable bacteria of Albanian subjects with different periodontal status compared to a similar population of Spanish subjects: a case control study
Source: BMC Oral Health. 2022 Mar 23;22:89. doi: 10.1186/s12903-022-02121-5 (PMC8944025; doi:10.1186/s12903-022-02121-5)
Supplement: Supplementary file 1 — Additional file 1. Presumptive identification of bacterial species in culture. [file 12903_2022_2121_MOESM1_ESM.docx]

**Supplementary Table S1** Presumptive identification of bacterial species in culture

**Table S1a** Bacteria *A. actinomycetemcomitans* and *E. corrodens* were identified by biochemical tests contained in the RapID ™ NH System KIT (REMEL OXOID-Thermo Fisher Scientific). Results are expressed as a series of positive percentages for each test of the system. Identifications were made based on test results on RapID NH panels along with other laboratory information (Gram stain, catalase, or growth on a differential or selective medium)

| **Bacterial species** | **Culture medium** | **Incubation** | **colony morphology** | **Gram-staining** | **catalase reaction** | **PRO** | **GGT** | **ONPG** | **GLU** | **SUC** | **EST** | **RES** | **PO4** | **ORN** | **URE** | **NO3** | **IND** |
| --- | --- | --- | --- | --- | --- | --- | --- | --- | --- | --- | --- | --- | --- | --- | --- | --- | --- |
| ***Aggregatibacter***  ***actinomycetemcomitans*** | Dentaid-1 | 3 days in air with 5% CO2 at 37ºC | Small colony, 1 mm in diameter, with a dark border and a “star” or “crossed cigars” shaped inner structure | negative | + | 46 | 98 | 1 | 95 | 16 | 59 | 39 | 58 | 1 | 0 | 79 | 0 |
| ***Eikenella***  ***corrodens*** | Blood Agar Base, with haemine, menadione and 5% sterile horse blood | 7-14 days of anaerobic incubation (80%N2, 10% CO2 and 10% H2) | Small and greyish, with a greenish discoloration of the underlying agar, with a typically depression (or “pit”) in the agar | negative | - | 86 | 0 | 9 | 0 | 0 | 0 | 23 | 0 | 97 | 0 | 93 | 0 |

PRO: Proline ρ-nitroanilide; GGT: γ-glutamyl ρ-nitroanilide; ONPG: σ-nitrophenyl, β, D-galactoside; GLU: Glucose; SUC: Sucrose; EST: Fatty acid ester; RES: Resazurin; PO4: ρnitrophenyl phosphate; ORN: Ornithine; URE: Urea; NO3: Nitrate; IND: Tryptophan

**Table S1b** Bacterial identification using biochemical tests contained in the RapID ™ ANA II System KIT (REMEL-OXOID-Thermo Fisher Scientific). Results are expressed as a series of positive percentages for each system test. Identifications are made based on test results on RapID ANA II panels along with other laboratory information (Gram stain, catalase, or growth on a differential or selective medium)

| **Bacterial species** | **Culture medium** | **Incubation** | **Colony morphology** | **Gram-staining** | **Catalase reaction** | **URE** | **BLTS** | **αARA** | **ONPG** | **αGLU** | **Β**  **GLU** | **αGAL** | **αFUC** | **NAG** | **PO4** | **LGY** | **GLY** | **PRO** | **PAL** | **ARG** | **SER** | **PYR** | **IND** |
| --- | --- | --- | --- | --- | --- | --- | --- | --- | --- | --- | --- | --- | --- | --- | --- | --- | --- | --- | --- | --- | --- | --- | --- |
| ***Porphyromonas gingivalis*** | Blood Agar Base, with haemine, menadione and 5% sterile horse blood | 7-14 days of anaerobic incubation (80%N2, 10% CO2 and 10% H2) | Small and round, smooth, shiny, convex with colour from green, reddish to brown | negative | - | 0 | 2 | 0 | 2 | 0 | 0 | 0 | 0 | 99 | 95 | 96 | 19 | 0 | 5 | 93 | 27 | 27 | 99 |
| ***Prevotella intermedia*** |  |  | Big shiny and smooth black pigmented colonies, rounded, flat and darker, with an irregular outline | negative | + | 0 | 0 | 0 | 0 | 99 | 0 | 0 | 93 | 0 | 98 | 98 | 4 | 0 | 0 | 96 | 6 | 0 | 99 |
| ***Tannerella forsythia*** |  |  | Pale pink and speckled, circular, convex, and may have a depressed centre (donut-shaped) | negative | - | 0 | 78 | 0 | 95 | 99 | 50 | 0 | 99 | 99 | 99 | 98 | 12 | 0 | 31 | 99 | 81 | 0 | 98 |
| ***Parvimonas***  ***micra*** |  |  | Small, rough or smooth, dome-shaped, bright white, with β-haemolytic halo colonies | positive | - | 0 | 0 | 0 | 0 | 4 | 0 | 0 | 0 | 5 | 93 | 95 | 98 | 92 | 88 | 98 | 96 | 86 | 0 |
| ***Fusobacterium nucleatum*** |  |  | Large, flat, irregular, whitish to pinkish and slightly grainy colonies | negative | - | 0 | 0 | 0 | 0 | 0 | 0 | 0 | 0 | 0 | 2 | 0 | 0 | 0 | 0 | 45 | 0 | 36 | 99 |
| ***Campylobacter rectus (*formerly *Wolinella* spp*.)*** |  |  | Small colonies, growing inside agar, translucent, slightly pink colour, round and convex. The center is higher than the rest, with shiny lines and points which are the limits of the “valley” | negative | - | 0 | 0 | 0 | 0 | 0 | 5 | 0 | 0 | 0 | 5 | 0 | 0 | 0 | 0 | 88 | 0 | 0 | 0 |
| ***Capnocytophaga* spp.** |  |  | Gliding and translucent reddish colonies | negative | - | 0 | 42 | 0 | 89 | 96 | 86 | 2 | 1 | 78 | 76 | 99 | 99 | 90 | 99 | 99 | 95 | 13 | 0 |
| ***Actinomyces odontololyticus*** |  |  | Small, irregular and convex colonies with dark centre (reddish) and white ring around it. They are called “bull-eyes” | positive | - | 0 | 4 | 15 | 86 | 96 | 46 | 5 | 92 | 78 | 76 | 99 | 99 | 90 | 99 | 99 | 95 | 13 | 0 |

URE: Urea; BLTS: ρ-nitrophenyl-β, D-disaccharide; αARA: ρ-nitrophenyl-α, L-arabinoside; ONPG: σ-nitrophenyl-β, D-galactoside; αGLU: ρ-nitrophenyl-α, D-glucoside; βGLU: ρ-nitrophenyl-β, D-glucoside; αGAL: ρ-nitrophenyl-α, D-galactoside; αFUC: ρ-nitrophenyl-α, L-fucoside; NAG: ρ-nitrophenyl-n-acetylβ, D-glucosaminide; PO4: ρ-nitrophenylphosphate; LGY: Leucyl-glycine-β-naphthylamide; GLY: Glycine-β-naphthylamide; PRO: Proline-β-naphthylamide; PAL: Phenylalanine-β-naphthylamide; ARG: Arginine-β-naphthylamide; SER: Serine-β-naphthylamide; PYR: Pyrrolidonyl-β-naphthylamide; IND: Tryptophan

**Table S1c** Additional tests in the identification of *P. gingivalis, P. intermedia, P. micra* and *T. forsythia*

| **Bacterial species** | **B** | **I** | **G** |
| --- | --- | --- | --- |
| ***Porphyromonas gingivalis*** | positive | positive | negative |
| ***Prevotella intermedia*** | negative | positive | positive |
| ***Parvimonas micra*** | negative | negative | negative |
| ***Tannerella forsythia*** | positive | negative | positive |

B: BANA test (N-α-Benzoil-DL-arginine-2-Naftilamide); I: Indole test (Kovacs reactive); G: α-Glucorunidase (β-naphthyl-α-D-glucopironidase)
